# Integrating Behavior of Children with Profound Intellectual, Multiple, or Severe Motor Disabilities With Location and Environment Data Sensors for Independent Communication and Mobility: App Development and Pilot Testing

**DOI:** 10.2196/28020

**Published:** 2021-06-07

**Authors:** Von Ralph Dane Marquez Herbuela, Tomonori Karita, Yoshiya Furukawa, Yoshinori Wada, Yoshihiro Yagi, Shuichiro Senba, Eiko Onishi, Tatsuo Saeki

**Affiliations:** 1 Department of Special Needs Education Graduate School of Education Ehime University Matsuyama, Ehime Japan; 2 Graduate School of Humanities and Social Sciences Hiroshima University Higashihiroshima, Hiroshima Japan; 3 Department of Contemporary Liberal Arts Faculty of Humanities and Social Sciences Showa Women's University Setagaya-ku, Tokyo Japan; 4 DigitalPia Co, Ltd Matsuyama, Ehime Japan

**Keywords:** profound intellectual and multiple disabilities, severe motor and intellectual disabilities, mobile app development, augmentative and alternative communication, AAC, smartphone-based data collection, behavior, child, sensor, communication, mobility, development, pilot, app

## Abstract

**Background:**

Children with profound intellectual and multiple disabilities (PIMD) or severe motor and intellectual disabilities (SMID) only communicate through movements, vocalizations, body postures, muscle tensions, or facial expressions on a pre- or protosymbolic level. Yet, to the best of our knowledge, there are few systems developed to specifically aid in categorizing and interpreting behaviors of children with PIMD or SMID to facilitate independent communication and mobility. Further, environmental data such as weather variables were found to have associations with human affects and behaviors among typically developing children; however, studies involving children with neurological functioning impairments that affect communication or those who have physical and/or motor disabilities are unexpectedly scarce.

**Objective:**

This paper describes the design and development of the ChildSIDE app, which collects and transmits data associated with children’s behaviors, and linked location and environment information collected from data sources (GPS, iBeacon device, ALPS Sensor, and OpenWeatherMap application programming interface [API]) to the database. The aims of this study were to measure and compare the server/API performance of the app in detecting and transmitting environment data from the data sources to the database, and to categorize the movements associated with each behavior data as the basis for future development and analyses.

**Methods:**

This study utilized a cross-sectional observational design by performing multiple single-subject face-to-face and video-recorded sessions among purposively sampled child-caregiver dyads (children diagnosed with PIMD/SMID, or severe or profound intellectual disability and their primary caregivers) from September 2019 to February 2020. To measure the server/API performance of the app in detecting and transmitting data from data sources to the database, frequency distribution and percentages of 31 location and environment data parameters were computed and compared. To categorize which body parts or movements were involved in each behavior, the interrater agreement κ statistic was used.

**Results:**

The study comprised 150 sessions involving 20 child-caregiver dyads. The app collected 371 individual behavior data, 327 of which had associated location and environment data from data collection sources. The analyses revealed that ChildSIDE had a server/API performance >93% in detecting and transmitting outdoor location (GPS) and environment data (ALPS sensors, OpenWeatherMap API), whereas the performance with iBeacon data was lower (82.3%). Behaviors were manifested mainly through hand (22.8%) and body movements (27.7%), and vocalizations (21.6%).

**Conclusions:**

The ChildSIDE app is an effective tool in collecting the behavior data of children with PIMD/SMID. The app showed high server/API performance in detecting outdoor location and environment data from sensors and an online API to the database with a performance rate above 93%. The results of the analysis and categorization of behaviors suggest a need for a system that uses motion capture and trajectory analyses for developing machine- or deep-learning algorithms to predict the needs of children with PIMD/SMID in the future.

## Introduction

### Background

Children with profound intellectual and multiple disabilities (PIMD) or severe motor and intellectual disabilities (SMID), as the name implies, have an estimated intelligence quotient of less than 25, which is equivalent to a maximum developmental age of 24 months [1,2]. These children often have difficulty in communication, especially understanding spoken or verbal language and symbolic interaction with objects [1,3]. The severe or profound motor disabilities are characterized by restricted or absence of hand, arm, and leg functions, resulting in limited or lack of ability to move independently [1,4]. In some cases, children with PIMD/SMID also have sensory impairments and chronic health conditions, which include but are not limited to epilepsy, visual impairments, constipation, spasticity, deformations, incontinence, and reflux [5,6]. Despite the severe challenges associated with these conditions, it is important to facilitate the ability of these children to communicate with people and interact with the environment independently.

Owing to their profound intellectual and neuromotor disabilities, one of the most challenging aspects of supporting children with PIMD/SMID is communication. Several augmented alternative communication apps have been developed that focus on helping children with speech disabilities, including a voice output communication aid (VOCA). With the help of mobile phones, VOCA apps such as Drop Talk and Voice4U have been helping children with speech disabilities communicate with other people. Their main function is to produce a voice when a user clicks a specific icon, symbol, or picture (display) that corresponds to a word or phrase. These displays can be combined (interface) to make sentences that can match a specific situation. Although VOCA offers a promising support approach for children with speech disabilities, selecting displays and choosing interfaces that best fit a specific situation are quite difficult tasks for children with speech and intellectual disabilities, because of their inability to determine which interface they should switch to in each situation and location due to their cognitive disability [1].

### Prior Work

In 2017, Karita [7] developed Friendly VOCA, a user-friendly VOCA iOS mobile app that enables children and individuals with speech and/or intellectual disabilities to communicate with other people independently. Unlike other available VOCAs, Friendly VOCA has the ability to automatically switch displays or interfaces that match the user’s location at a specific time [7]. To achieve this, Friendly VOCA uses GPS technology to identify the user’s current outdoor location in terms of map coordinates (latitude and longitude). However, to address the inability of GPS to identify indoor locations (eg, inside a store or a room) and elevated surfaces (eg, building floors), iBeacon was utilized. iBeacon is a system developed by Apple Inc that is based on Bluetooth low energy (BLE) proximity sensing, which transmits a universally unique identifier and radio signal strength indication (RSSI) to a user’s app. These two combined systems have helped Friendly VOCA to switch interfaces, which are displayed automatically depending on the user’s location at a specific time. Both the GPS and iBeacon systems have been tested, and experiments revealed that they can automatically show appropriate interfaces and displays that correspond to users’ locations with 100% and 71% accuracy, respectively [7].

### Script Theory and Location Data

Grounded in Schank and Abelson’s [8] script theory, Friendly VOCA’s concept of automatically switching displays or interfaces that match the user’s location is based on the notion of “scripts.” Scripts are the organized set or body of our basic background knowledge or “schema” that we must have to understand how we respond or behave appropriately to a particular situation or location [8]. This theory was used to structure the schema of Friendly VOCA on specific scripts in the form of varied displays and interfaces tailored to a specific situation (eg, class or playtime), location (eg, classroom, playground, home), and time (eg, morning, lunch breaks, evening) using the GPS and iBeacon systems [7].

Although the use of scripts greatly matches the intention of Friendly VOCA, it may also present possible misunderstandings or incorrect inferences due to many variations of situations or locations (eg, type of restaurant), where a general script may not be applicable (eg, different scripts in fast food and fine dining restaurants). Similarly, Friendly VOCA’s set of displays and interfaces may not perfectly cater to all children with speech and/or intellectual disabilities since each child has personalized needs that are beyond the abilities that Friendly VOCA can currently provide. Most importantly, it also neglects the specific needs of children with PIMD/SMID. Since Friendly VOCA requires the user to choose and click an icon or symbol to produce a voice output, apparent understanding of symbolic interaction (interpreting symbols or icons) or verbal language (comprehending voice outputs) is required, which may seem difficult for these children due to their severe or profound intellectual disabilities [1]. For example, children with PIMD/SMID may not understand that a symbol or a picture showing a hand with its index finger pointing to a face means “I,” “I am,” or “me,” let alone understand the meaning of the voice output that corresponds to the symbol. Moreover, clicking an icon or symbol can also be physically demanding for some children with profound neuromotor dysfunctions [1].

### Relation of Environment Data With Children’s Behavior and Affect

Environment data such as weather variables, including humidity, wind speed, precipitation, decreased visibility, and less hours of daylight, were found to have associations with human affects and behaviors such as physical activities among typically developing children [9-12]. Yet, similar studies involving children with neurological functioning impairments that affect communication or those who have physical or motor disabilities are unexpectedly scarce. VanBurskirk and Simpson [13] investigated the relationship between meteorological data (ie, barometric pressure, humidity, outdoor temperature, and moon illumination) with classroom-collected behavioral data of three children with autistic disorders who had significant behavior problems, including screaming, falling to the floor, head-butting, biting, kicking, hitting, and elopement. In contrast with the results of similar investigations among typically developing children, there was a weak relationship found between the behavior patterns demonstrated by the children with autism and meteorological parameters [13]. Notably, the selection of weather variables was only based on previous studies among typically developing children, since related studies on children with autism had not been performed. Most importantly, given the fact that the study was in its initial stage with clear methodological limitations, the authors stressed that the results must be interpreted with caution and should be further investigated, which might yield different results among children with more nuanced behavior [13].

### Categorizing the Behaviors of Children With PIMD/SMID

Children with PIMD/SMID only communicate through movements, sounds, body postures, muscle tensions, or facial expressions on a presymbolic (nonsymbolic) or protosymbolic (limited information) level with no shared meaning, which hinders expressing their needs [14-17]. These behaviors can also be minute and refined, which may be difficult for caregivers and teachers to perceive and interpret their needs [14]. Surprisingly, to our knowledge, prior to the studies of Tanaka et al [18], Motoda et al [19], and Ashida and Ishikura [14,20], scarcely any study had examined the behaviors of children with PIMD/SMID to enable perception and interpretation. In 2013, Ashida and Ishikura [14] introduced six major categories based on the body parts movements involved in each expressive behavior of children with PIMD/SMID: eye movement, facial expression, vocalization, hand movement, body posture, body movement, and noncommunicative behaviors (others). They then used these categories to analyze the expressive behaviors of two children in 2015 [20]. They found that one child had many active movements of the arms, legs, and eyes, and expressed their needs and emotions by changing gaze and smiling, whereas the expressions of the other child were limited to the movements of the head, neck, mouth, and eyes [20]. This suggests that to predict the needs of children with PIMD/SMID, interventions that focus on interpreting their behaviors, whether they involve head, face, or upper limb movements, can be developed. However, to realize this goal, it is first necessary to collect data on the children’s behaviors associated with their needs. Yet, to the best of our knowledge, there is hardly any technology specifically developed for this purpose.

### Mobile-Based Data Collection

The smartphone is now widely used as a data collection tool in psychological studies [21]. Its use has also advanced field experiment methodology such as broadening the scope reach, control randomization, and ability to collect a wide variety of data over time through the use of mobile apps [22]. Smartphone-based data collection through the use of apps provides real-time data, and is an efficient and accurate method with minimal errors and inconsistencies [23,24]. Apps, through a user interface, can also combine data available in smartphones (eg, GPS) or other mobile-sensing devices (usually using Bluetooth technology) to facilitate collection (frontend), which can be transmitted through the portal server and stored in a database (eg, MySQL, Google Firebase) (backend), and extracted for data processing during or after interventions [21,24]. Integrating location and sensor data can provide more fine-grained studies of behavior expression across situations and behavior inference [21,25]. Data collected from mobile apps and sensors are usually used to extract useful features to build a predictive model with machine-learning algorithms [26]. Thus, we developed ChildSIDE, a mobile app that collects behavioral data from children with PIMD/SMID as interpreted by their caregivers. Similar to Friendly VOCA and grounded in the notion of scripts, the ChildSIDE app also collects location and environment data through the use of location and environment (weather) data-sensing technologies and an online application programming interface (API). By not only collecting and analyzing children’s behaviors but also collecting and analyzing location and environment data associated with each behavior, ChildSIDE could help to infer their intentions and needs in the future.

### Goals and Hypotheses of This Study

This paper describes the design and development of the ChildSIDE app. The app was pilot-tested among purposively recruited children with PIMD/SMID and their caregivers, and its server/API performance was investigated in terms of detecting and transmitting location and environment data to the app database. Another aim of this study was to identify which movements were associated with the children’s behaviors by categorizing the movements using the table of expressions proposed by Ashida and Ishikura [14]. This will help in identifying the method or design of the system that will be further developed in the future. This study is exploratory in the context of testing the app’s server/API performance in detecting and transmitting environmental data using the sensors, API, and outdoor location (GPS), but not the use of the iBeacon system for indoor location due to the relatively low server/API performance rate of iBeacon based on a previous experiment [7]. According to previous literature, we hypothesized that the children’s behavior will mainly involve head, face, or upper limb movements.

## Methods

### App Design, Development, and Interface

ChildSIDE, a mobile app, was developed to collect: (a) caregivers’ interpretations of the behaviors of children with PIMD/SMID, (b) location, and (c) environment data. The app was developed on the Android (OS Android 6.0) mobile platform (HUAWEI P9 lite; Kirin 650 Octa Core, 4×2.0 GHz and 4×1.7 GHz) using Eclipse Android Studio (version 4.0.1), an integrated development programming environment software, and Java 1.80_242 (OPEN JDK) programming language in Windows 10 Pro (1909) [27,28]. The design of ChildSIDE was based on internet-of-things systems for a human-computer interaction interface and its name originates from the main goal of being “beside” its target population, children (“Child”), by aiding independent communication and mobility. “SIDE” is also an acronym for “Sampling Information and Data of children’s expressive behaviors and the Environment,” which is explicitly derived from its main function of collecting children’s behaviors with associated location and environment data. The completed app used in the pilot-testing sessions was installed on two Huawei Nova lite mobile phone (OS EMUI 8.0 based on Android 8.0) with a HUAWEI Kirin 659 Octa Core CPU (4×2.36 GHz + 4×1.7 GHz) [29].

**Figure 1:**
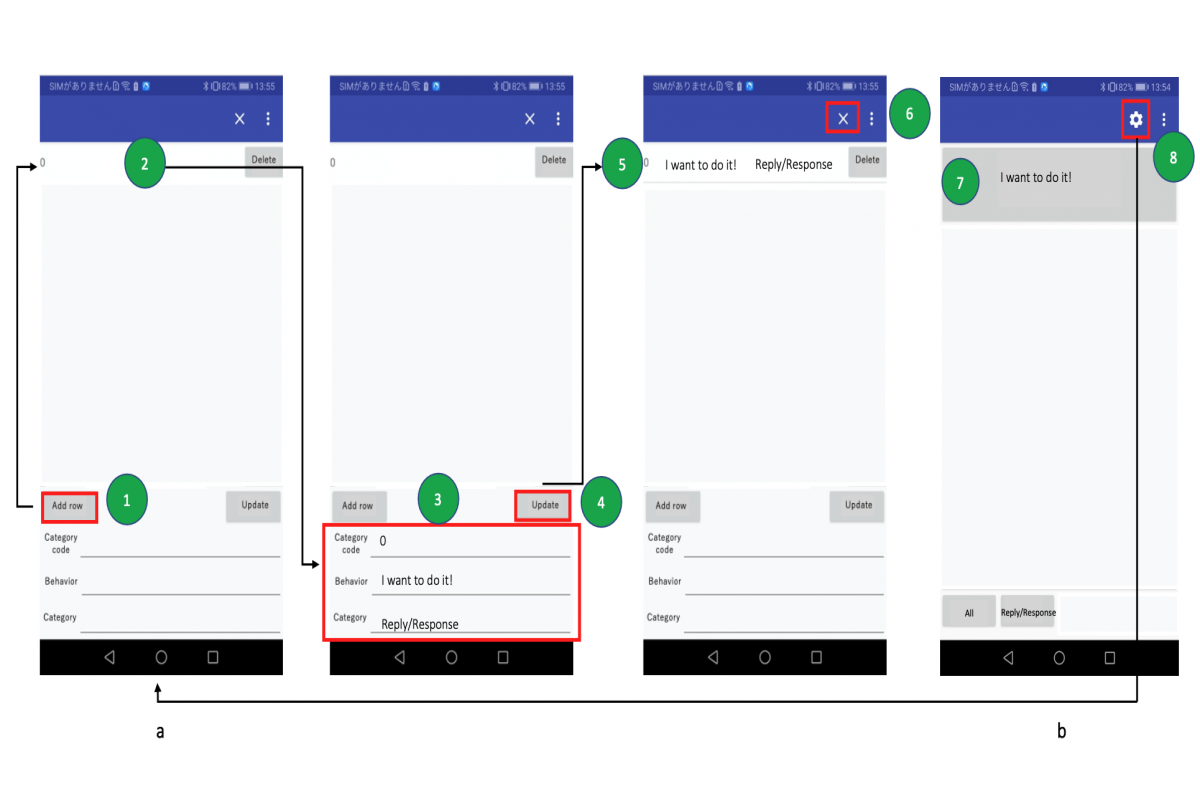
ChildSIDE user interface and guide. (a) Behavior settings, (b) behavior list.

ChildSIDE has two interfaces (Figure 1): a behavior settings interface (a) and a behavior list interface (b). The behavior settings interface allows the user to add a behavior. Users should click the “Add row” button to add a new row, and then a new row will appear in the list above it (2). The user can then enter the category code, the behavior’s name, and a category name in the settings interface below this new row (3). The assigned codes correspond to the order of behaviors the user wants to appear in the behavior list interface (b). If the user wants to put the most common behavior at the top of the list, the code should be 0, and the second most common behavior is coded “1,” which follows the behavior that was coded 0, continuing in this manner. To save the information, users should click the “update” button (4), and then the new behavior with its corresponding code and category name will appear in the list above on the setting interface (5). The “x” button (6) on the upper right corner of the interface should be clicked to go to the behavior list interface (b). The behavior list interface shows the behavior name on the top row and the category name in a space below. When a user clicks a behavior name (7), the app automatically sends the behavior and category name with its associated location and environment data to the database. To add or edit a behavior, the user needs to click the gear button (8) on the upper right corner of the interface to go to the behavior settings (a). When adding a new behavior to an already existing category, users need to enter the name of the category; otherwise, a new category will be created. Categorizing the behaviors will make it easier for the user to locate or update them subsequently.

### Location and Weather Data Sources

**Figure 2:**
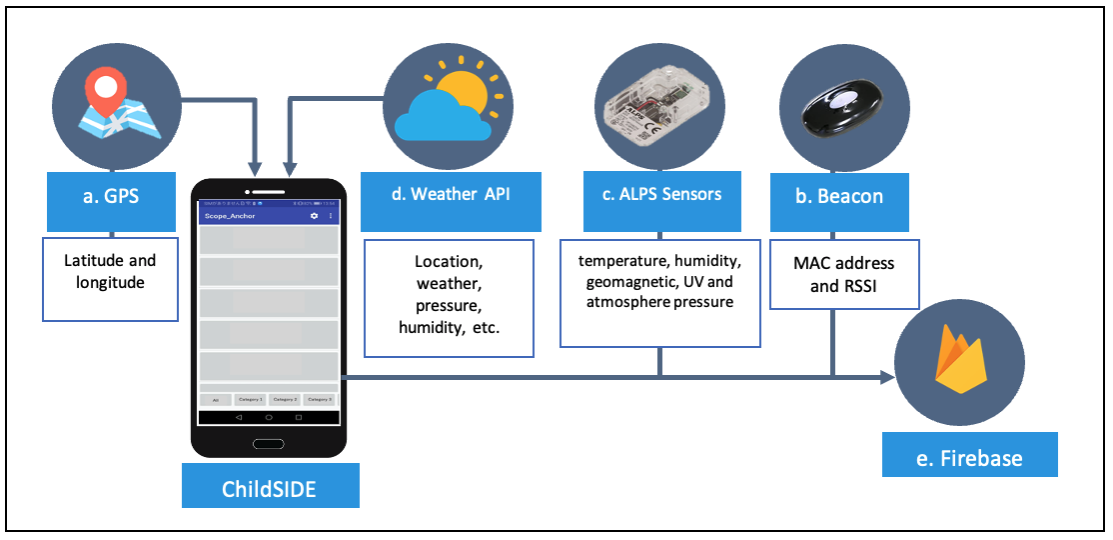
Data flow from the data sources (iBeacon, GPS, ALPS Sensors, and OpenWeatherMap API) detected and transmitted by ChildSIDE app to the Google Firebase database. API: application programming interface; RSSI: radio signal strength indication.

Figure 2 shows how the ChildSIDE app collects data from the data sources (iBeacon, GPS, ALPS Sensors, and OpenWeatherMap API) and transmits the data to the Google Firebase database. The Android’s built-in time stamps and GPS (GPS/AGPS/Glonass) (a) were used to identify the user’s current outdoor location in terms of map coordinates (latitude and longitude) [29].

iBeacon (Figure 2, b) (BVMCN1101AA B), from module BVMCN5103BK manufactured by Braveridge [30], has a 2402 to 2480 MHz frequency range, –20 to +4 dBm transmission power (terminal output), is AA battery-powered, and operates on 2.2 volts [30]. iBeacon is based on BLE proximity sensing that provides proximity-based app services, and coarse-grained indoor location positioning and navigation [31]. iBeacon has widespread applications that range from advertisement (providing product information), education (interactive activities for museum visitors), and tracking (luggage at the airport or patients in emergency rooms) to an evacuation guide system during emergency situations [31]. It transmits proximity measurements based on RSSI and MAC address (6 bytes: F5:B0:E2:A2:AE:69), and uses an iBeacon name to a close mobile device to identify the user’s specific indoor location [32]. The RSSI is the strength of the beacon’s signal relevant to the receiving device that determines the distance between them, which ranges from –26 to –100 (in inches) [33]. The transmission accuracy between the mobile device and iBeacon can be categorized as immediate (0 to 0.5 meters), near (0.5 to 2 or 3 meters), or far (2 or 3 meters to 30 meters) [32,33]. The Bluetooth LE model of Braveridge BVMCN1101AA B is certified by the Bluetooth special interest group, Japan Radio Law (Japan Quality Assurance Association), and is US Federal Communications Commission (FCC) Part 15-compatible and Harmonized European Standard EN300 328 (ETSI Technical Committee electromagnetic compatibility and radiospectrum matters)-compatible [30].

The IoT Smart Module Sensor Network Module Evaluation kit consists of a multifunction Bluetooth sensor (ALPS Sensor; Figure 2, c) module (Mouser and manufacturer number: 688-UGWZ3AA001A Sensor Network Kit W/BLE Mod Sensors) developed by ALPS Alpine [34]. It has a 2.4 GHz frequency, operates with a 3.3 volt supply, and has –93 dBm Bluetooth receiver sensitivity [34]. It includes multiple sensors for pressure, temperature, humidity, ultraviolet, ambient light, and 6-axis (Accel + Geomag); it also has a built-in microcontroller unit or processor (memory and input/output peripherals on a single chip) for realizing efficient power management and an ultracompact module realized with high-density mounting technology [34]. The kit is used to monitor work environments, and in fitness and health care [34]. It has also been used to acquire and transmit 11 motion and environment data: temperature (°C) and relative humidity (RH%) geomagnetic sensor (electric compass; 6-axis Accel+Geomag; ranges: g1, g2, g3, and resolutions μT1, μT2, μT3); ultraviolet or ambient light (mW/cm^2^ and Lx), and atmospheric pressure (hPa) [34].

Weather data (atmospheric pressure, humidity, sunrise and sunset time) were obtained from OpenWeatherMap API (Figure 2, d), an online service that provides weather data that matches the user’s current location [35]. OpenWeatherMap API uses a numerical weather prediction model (90% and 100% reliability, and 1% inaccuracy) from several data sources (global: NOAA GFS 0.25 and 0.5 grid sizes, NOAA CFS, ECMWF ERA; weather stations: METAR stations, users’ stations, companies’ stations; and weather radar data and satellite data) in 371 national capitals and major cities [35]. The API has 15 parameters: country name, location name (region or city), weather, sunset time, sunrise time, current time, minimum temperature (°C), maximum temperature (°C), atmospheric pressure (hPa), main temperature (°C), humidity (%), weather description, cloudiness (%), wind direction (degrees), and wind speed (meters/second) [35]. When a user clicks a behavior, the app automatically sends the behavior and category name with its associated GPS and iBeacon location data, and environment data from the OpenWeatherMap API and the ALPS sensors to the Google Firebase database (Figure 2, e), a third-party service provider that allows the data to be stored in real time and synchronized among mobile platforms [36].

As previously mentioned, weather variables, in particular humidity, solar radiation, wind speed, visibility, hours of daylight, barometric pressure, temperature, and moon illumination, were found to have association with the emotions and behaviors of typically developing children [9], yet little is known about these effects among children with neurological or physical impairments, specifically among children with PIMD/SMID. Thus, since the majority of these studies, including this study, were exploratory and had no specific inclusion criteria on what weather parameters should be investigated, all weather parameters of ALPS Sensors and OpenWeatherMap API were included. Most importantly, most of the weather variables investigated in previous studies are similar to those collected by ALPS sensors and OpenWeatherMap API.

### Study Design, Sampling, and Participant Inclusion Criteria

For pilot testing, we utilized a cross-sectional observational study design by performing multiple single-subject face-to-face and video-recorded sessions. Studies that used a single-subject design among children with special education needs showed more powerful results than those that used a group research design [37]. The app was pilot-tested among purposively sampled child-caregiver dyads recruited at a special needs school from September 24, 2019 to February 25, 2020.

The children included in this study met the following criteria: diagnosed with PIMD/SMID or severe or profound intellectual disability, with or without comorbid sensory impairments and/or chronic health conditions, which include but are not limited to epilepsy, visual impairments, constipation, spasticity, deformations, incontinence, and reflux, and with a chronological or mental age of 18 years and below at the time of the study. Caregivers were either the primary (immediate family members) or secondary (nonfamily, including teachers, supporters) caregivers who had been living or supporting the children for 3 years or more. This criterion was set to ensure that caregivers were familiar and have a working schema about the children’s behaviors.

### Ethical Considerations

This study was part of a project that was written, performed, and approved as per international ethical guidelines (Declaration of Helsinki [38] and the International Council for Harmonization Good Clinical Practice guidelines, approval number: R2-18) [39]. The parents or caregivers of all participants provided their consent for the child’s participation in this study by signing a written informed consent form. They were also informed that their participation in the study was voluntary and that they may stop their participation at any time. All data that contain participant information or identity were coded and blurred, respectively, and are stored in a password-protected network server database and computer for their protection and privacy.

### Intervention

#### Experimental Setup

**Figure 3:**
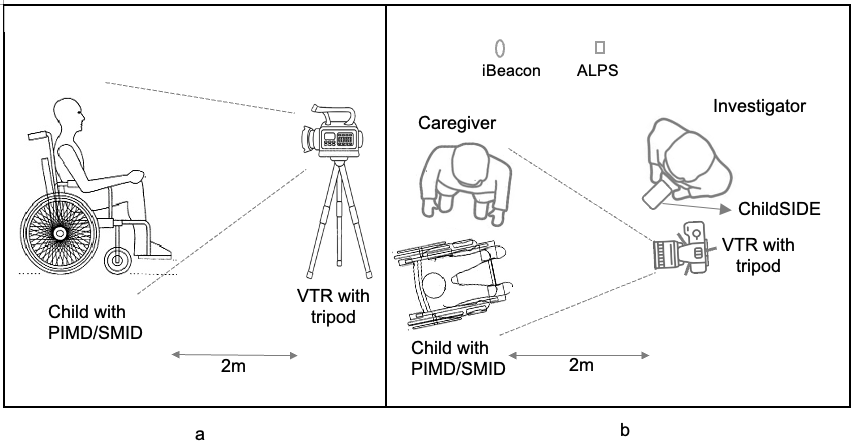
Intervention setup. (a) Videotape recorder (VTR) focusing on facial, and upper and lower limbs movements. (b) Intervention setup in a classroom setting: 2-meter distance from the VTR to the child with profound intellectual and multiple disabilities (PIMD) or severe motor and intellectual disabilities (SMID) and caregiver, and the location where the iBeacon and ALPS sensors were placed.

**Figure 4:**
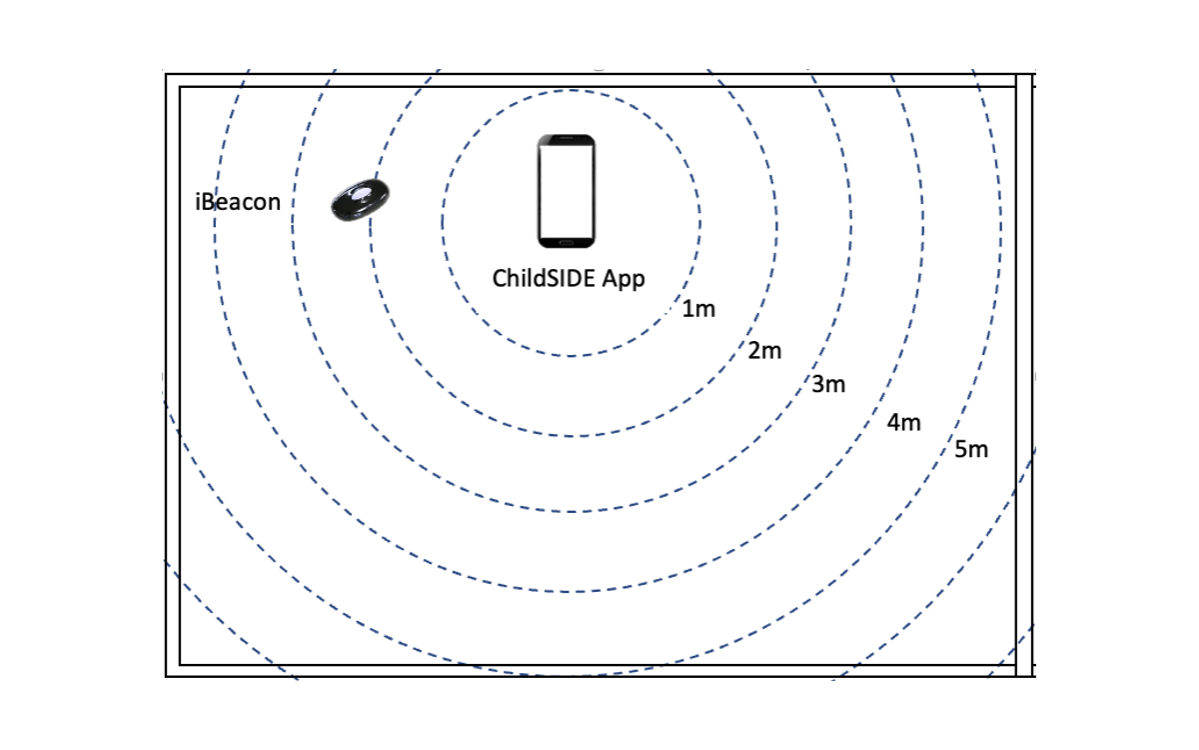
A sample experimental setup showing how the radio signal strength indication of iBeacon is detected by the ChildSIDE app with an actual distance of 2 meters.

A video-based recording method was used in all sessions for interrater analyses and categorizing of behaviors. This method has been reported to have higher interrater reliability than traditional observational methods, and allows researchers to collect, analyze, and validate data retrospectively [40]. One videotape recorder in a tripod placed 2 meters from the participants was used to capture the child’s facial expressions, and upper and lower limb movements (Figure 3a), and all exchanges of responses between the child and their caregiver (Figure 3b). Before the sessions, one iBeacon device and one ALPS sensor were installed in each location (there were a total of 18 different locations that included classrooms, music room, playrooms, and others). They were installed either on a shelf, blackboard, bulletin board, or on an air conditioning unit with an approximately 2-meter distance (estimated mean distance 2.18 meters, SD 0.09, mean error 0.184, root mean square error 0.411) from the ChildSIDE app (Figure 4). For more information on the location (room and specific location installation), sampling frequency, estimated mean distance, SD, mean error, and root mean square error of each iBeacon device used in the sessions, please refer to Tables S1 to S3 in Multimedia Appendix 1.

#### Sessions

Multiple single-subject face-to-face sessions were performed; the duration of each session depended on the child’s availability and the willingness of their caregivers to participate and be recorded. The sessions were divided into three batches (batch session 1, elementary; batch session 2, junior high school; batch session 3, high school). The three batches of sessions were performed within 16 days with an average of 4.2 sessions per day (SD 1.95). In total, there were 105 sessions performed that ranged from as few as 1 session and as many as 15 sessions per child (with an average of 5 sessions per child). Initially, we performed 90 sessions (recording time range 0.37-32 minutes, mean 19 minutes, SD 11.3 minutes among 19 children). When another child was recruited, we still performed 15 additional sessions (recording time range 6 to 54 minutes, mean 28 minutes, SD 13.8 minutes) among the participants.

All sessions were recorded in the locations where the children usually spend time to ensure they can behave normally and interact with their caregivers even in the presence of other children, caregivers, and an investigator. The sessions did not interfere with the academic lessons where children interact more with the main teacher or supporter. Initially, the investigator identified the behaviors under investigation based on the categories and description of the body parts and movements involved in the behavior of children with PIMD/SMID. However, since our aim was to collect behaviors linked to the children’s specific needs, the investigator frequently had to add new behaviors as required, especially when a child exhibited a certain reaction (eg, vocalization, gesture) and when the caregiver responded by confirming the child’s need (eg, want to go to the toilet) verbally or by actions (eg, assist the child to the toilet). The sessions targeted their behavior during morning greetings, lunchtime, and break time when they would always interact more with their caregivers who attended to their needs.

To control for the confounding effect of the presence of an investigator or the intervention, the investigator was trained and was instructed to strictly prevent interacting with the participants or interfering with the sessions. All sessions were performed by only one trained investigator with the expectation that the participants will be familiar and comfortable with the session procedures and with the investigator to ensure collecting fairly consistent and valid behavior data from the participants.

### Statistical and Data Analysis

#### Server/API Performance

To measure server/API performance of ChildSIDE in detecting and sending data from data sources to the database, frequency distribution and percentages of the 31 location, motion, and environment data types from each data source were computed, including 3 from iBeacon, 2 from GPS, 11 from ALPS sensors, and 13 from OpenWeatherMap API. The behavior data without any associated data from any data source were deleted. Each transmission to the app database was scored “1” and errors (ie, app failed to detect signals from sensors or vice versa) were scored “0.” Since each data source has multiple data types, the mean scores were computed and used for comparison to the total number of behavior data with associated location and environment data.

#### Interrater Agreement

To categorize the body parts or movements (minor categories) involved in each behavior using the table of expressions in children with PIMD/SMID (Table 1) [14], two raters watched the video recordings independently and analyzed each behavior recorded by the app. Each rater provided a score of “1” in each minor category to which a behavior belongs; otherwise, a score of “0” was given. For example, “Goodbye” can be expressed by waving the hands and producing sound; thus, this behavior was given a score of 1 for the minor category “moving” under the major category hand movement (Table 1, d.3) and a score of 1 for the major category vocalization (Table 1, c). To test the agreement between the two raters in each behavior per minor and major category, κ statistics were computed. To identify the κ coefficients in each major category, each category was given a score of 1 when there was at least one minor category with a score of at least 1. The level of agreement was assessed as follows: κ=0, less than chance; κ=1.01-0.20, slight; κ=0.21-0.40, fair; κ=0.41-0.60, moderate; κ=0.61-0.80, substantial; and κ=0.81-0.99, almost perfect agreement.

Interrater agreement between the two raters in each major and minor category was assessed according to κ coefficients with a significance level of *P*<.01 [41]. The two raters also counted the number of times (frequency) each movement (minor category) was displayed for each behavior. Lastly, the raters reanalyzed their responses, and once a consensus was reached, a final categorization of behaviors was created based on the table of expressions in children with PIMD/SMID. All statistical analyses (χ^2^ and κ) were performed using the “stats” (version 4.0.1) and “irr” (version 0.84.1) packages of R (version 4.0.2) statistical computing software.

**Table 1:** Category table of expressions in children with profound intellectual and multiple disabilities or severe motor and intellectual disabilities [14].

Categories		Criteria
**a. Eye movement**		
	1. Gazing	Gaze at people and things (in the case of people, look at their faces)
	2. Eye tracking	Eye movements that follow the movements of people and things in a linear fashion
	3. Changing line of sight	Change of line of sight, movement of line of sight; gaze rolls and moves; point-like movement that is distinct from “a.2. eye tracking.” The momentary glare can also be evaluated. Movements that cannot be evaluated as gaze/tracking
	4. Opening or closing the eyelids	Not an involuntary blink; their reaction when told to open or close their eyes
**b. Facial expression**		
	1. Smiling	Smile
	2. Facial expression (other than smile)	Something that is not expressionless; changes in facial expressions (eg, surprise, frowning, sticking out tongue)
	3. Concentrating and listening	Focusing on picture books, music, and voices etc
c. Vocalization		Producing sound
**d. Hand movement**		
	1. Pointing	Hand pointing or pointing finger toward an object
	2. Reaching	The action of reaching or chasing after reaching the target, not by pointing the hand or finger
	3. Moving	Grab, hit, beckon, push, raise hands, dispel, etc
**e. Body movement**		
	1. Approaching	Head or upper body (or the whole body) is brought close to a person or an object
	2. Contacting	Touching people and things with the hands and body; excludes cases that are touched by accident
	3. Movement of a part of the body	Head and neck movements, upper body movements, upper and lower limb movements (eg, shake, bend, move mouth, flutter legs); excluding “d.1. pointing,” “d.2. reaching,” “d.3. moving,” and distinguished from “f.1. stereotyped behavior”
**f. Noncommunicative behaviors (others)**		
	1. Stereotypical behavior	The same behavior or movement is repeated without purpose; behavior that occurs in a certain repetition (eg, finger sucking, shaking hands, rocking), excluded from shaking things in “d.3. moving”
	2. Injurious behavior to self and others	Hitting someone, biting finger, etc
	3. Others	Difficult to classify other than the above categories

## Results

### Participants Profile and Session Outcomes

A total of 19 of 22 child-caregiver dyads (3 dyads were excluded owing to unavailability) were assessed for eligibility. The children were aged from 8 to 16 years (equivalent of 3rd grade to 1st year of high school), 13 (68%) were males, 15 (79%) had PIMD/SMID diagnoses, and 4 (21%) had severe or profound intellectual disabilities.

**Figure 5:**
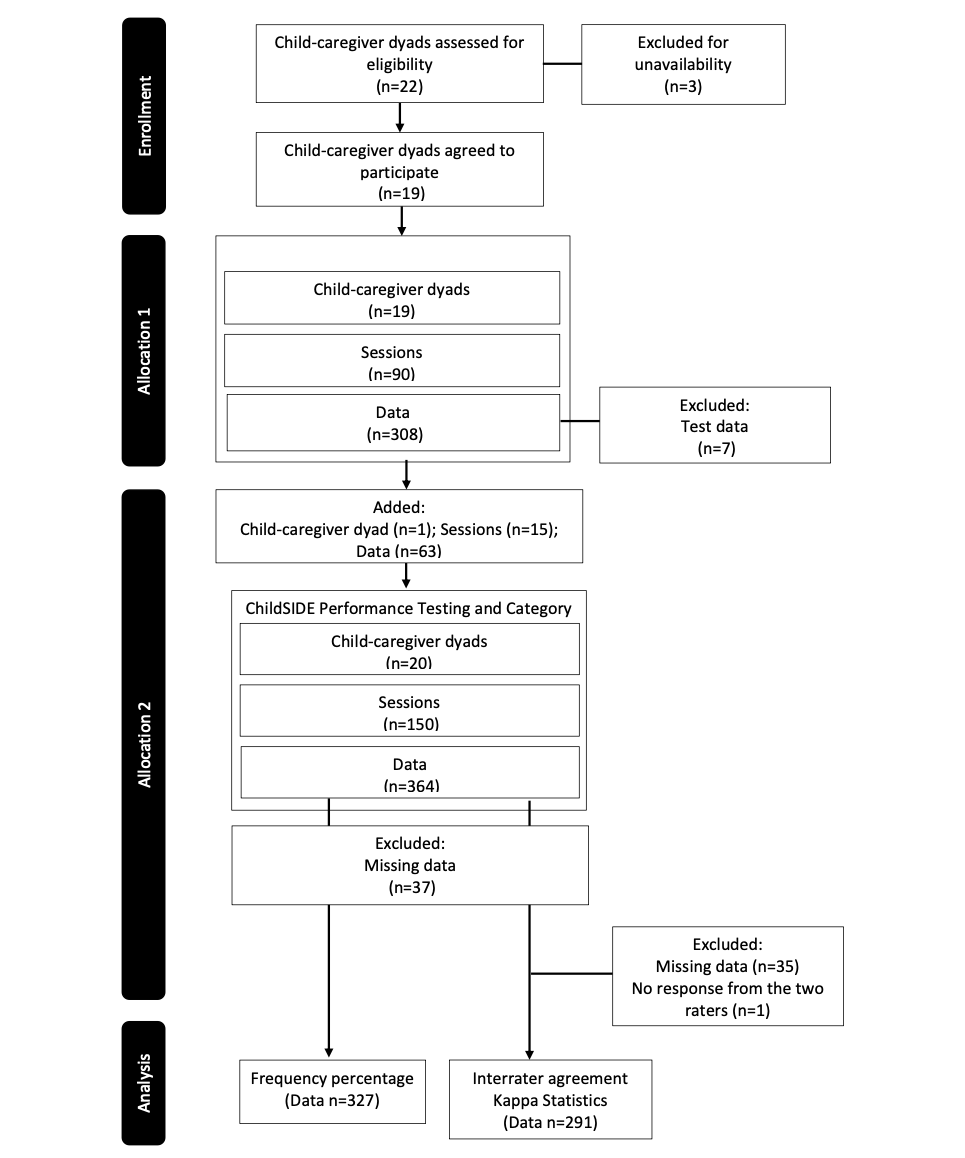
CONSORT diagram of participant, session and data flow from enrollment, allocation, and analysis.

Figure 5 shows the participant, session, and data flow using the CONSORT diagram. In the 16-day collection period, 90 sessions were performed wherein 308 individual behavior data were collected. The daily average data collected was 20 (SD 12.2) and the average number of data collected per session was 5.2 (SD 2.2). Seven of these were identified as test data, which were excluded, bringing the total number to 301. From the 19 child-caregiver dyads, one child-caregiver dyad (8-year-old male with PIMD/SMID) was added, leading to the addition of 15 additional sessions and 63 individual behavior data, bringing the total to 20 child-caregiver dyads, 150 sessions, and 364 individual behavior data collected. Of the 364 individual behavior data, 37 without any associated data from any data source were deleted. In total, 327 data entries with associated data from iBeacon, GPS, ALPS, Sensor, or OpenWeatherMap API data sources were used to compare frequency percentages. In the interrater agreement assessment used for categorizing the behavior data, 35 individual behavior data that were not detected by the app were also deleted. From the total 292 individual behavior data (see Multimedia Appendix 2 for more information on the individual behavior data), one had no score from the two raters, subjecting only the remaining 291 to interrater agreement (κ) statistical analysis.

### iBeacon, GPS, ALPS Sensor, and OpenWeatherMap API data

**Figure 6:**
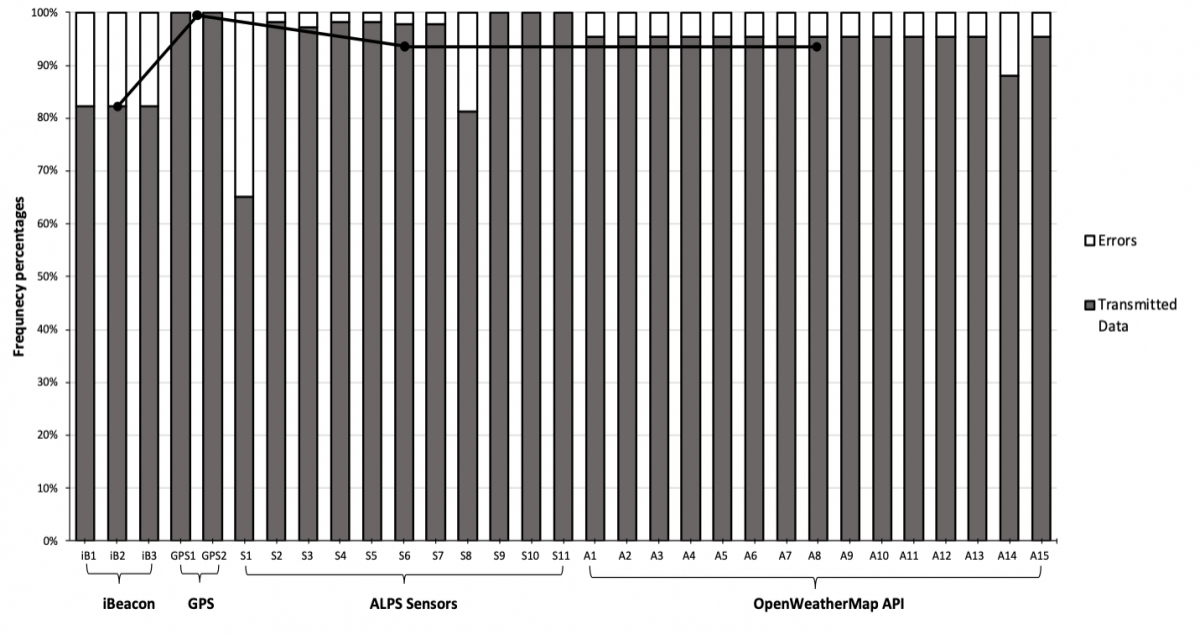
Frequency percentages of transmitted data and errors in each data type, and mean scores (black dots) in each data source detected and transmitted by the ChildSIDE app to the database. iB1: MAC address; iB2: radio signal strength indication; iB3: iBeacon name; GPS1: longitude; GPS2: latitude; S1: ultraviolet (UV) range (mW/cm2); S2, S3, S4: 6-axis (Accel+Geomag) sensor ranges [g]; S5, S6, S7: 6-axis (Accel+Geomag) sensor resolutions [μT]; S8: UV resolution [Lx]; S9: pressure sensor range (hPa); S10: temperature and humidity sensor range (℃); S11: temperature and humidity sensor resolution (%RH); A1: country name; A2: location name (region or city); A3: weather; A4: sunset time; A5: sunrise time; A6: current time; A7: minimum temperature (℃); A8: maximum temperature (℃); A9: atmospheric pressure (hPa); A10: main temperature (℃); A11: humidity (%); A12: weather description; A13: cloudiness (%); A14: wind direction (degrees); A15: wind speed (meters/second).

The app was able to detect and transmit 269 (82.3%) MAC addresses, 269 (82.3%) RSSI, and 269 (82.3%) iBeacon names (Figure 6). Ultraviolet or ambient light sensors range (S1 in Figure 6) and resolution (S8 in Figure 6) had the relatively lowest scores at 213 (65.1%) and 266 (81.3 %), respectively, among the ALPS sensors. The scores for the 6-axis (Accel+Geomag) sensor ranges (S2, S3, and S4 in Figure 6) and resolutions (S5, S6, and S7 in Figure 6) ranged from 318 (97.2%) to 321 (98.2%). Among the ALPS sensors, 100% of the pressure sensor range (S9 in Figure 6), temperature and humidity sensor range (S10 in Figure 6), and resolution (S11) data were detected and transmitted by the app to the database. Among the OpenWeatherMap API parameters, wind direction (A14 in Figure 6) had a relatively lower score of 288 (88.1%) compared with that of the other parameters (A1 to A13 and A15 in Figure 6) that had scores of 312 (95.4%). In general, iBeacon had the relatively lowest mean score (269, 82.3%) among the data sources: GPS (327, 100%), ALPS Sensors (305, 93.4%), and OpenWeatherMap API (310, 94.9%). This means that the ChildSIDE app shows a server/API performance ranging from 82% to 100% in detecting and transmitting location and environment data to the database.

### Behavior Categories

Table 2 shows the levels of agreement based on the κ coefficients and range between the two raters in identifying the body parts or movements (minor categories) involved in each behavior. The κ statistics revealed that the levels of agreement between the two raters in 14 out of 16 minor categories ranged from fair (κ=0.21-0.40) to almost perfect (κ=0.81-0.99) with statistical significance (*P*<.001).

The minor categories with the highest and lowest κ coefficients were pointing and stereotypical behaviors with κ coefficients of 0.88 and 0.21, respectively. Only one rater scored a need under the concentrating and listening category, and the behaviors that fell under the injurious to self and others category differed between the two raters. Further, although the two raters had an almost perfect level of agreement (*P*<.001) in vocalization (0.95) and hand movement (0.88), and substantial level of agreement in eye movement (0.83), facial expression (0.70), and body movement (0.78), noncommunicative behaviors (others) only had a κ coefficient of 0.40, representing a fair interrater agreement level. From these results, we were able to identify 676 body parts or movements involved in 291 individual behavior data. Of these 676, children’s behaviors comprised 27.7% body movement, 22.8% hand movement, 21.6% vocalization, 15.4% eye movement, 9% facial expression, and 3.6% other expressions.

**Table 2:** Interrater agreement and frequency distribution of the major and minor categories of the table of expressions in children with profound intellectual and multiple disabilities or severe motor and intellectual disabilities [14].

Categories			Interrater agreement						Frequency distribution (N=676), n (%)
			κ		κ range^a^	*P* value			
**a. Eye movement**			0.83		5	<.001		104 (15.4)	
	1. Gazing	0.64		4		<.001	38 (5.6)		
	2. Eye tracking	0.50		3		<.001	13 (1.9)		
	3. Changing line of sight	0.53		3		<.001	46 (6.8)		
	4. Opening or closing the eyelids	0.74		4		<.001	7 (1.0)		
**b. Facial expression**			0.70		4	<.001		61 (9.0)	
	1. Smiling	0.69		4		<.001	36 (5.3)		
	2. Facial expression (other than smile)	0.34		2		<.001	24 (3.6)		
	3. Concentrating and listening^b^	N/A^c^		N/A		N/A	1 (0.1)		
c. Vocalization			0.95		5	<.001		146 (21.6)	
**d. Hand movement**			0.88		5	<.001		154 (22.8)	
	1. Pointing	0.88		5		<.001	29 (4.3)		
	2. Reaching	0.69		4		<.001	25 (3.7)		
	3. Moving	0.79		4		<.001	100 (14.8)		
**e. Body movement**			0.78		4	<.001		187 (27.7)	
	1. Approaching	0.44		3		<.001	16 (2.4)		
	2. Contacting	0.76		2		<.001	35 (5.2)		
	3. Movement of part of the body	0.64		2		<.001	136 (20.1)		
**f. Noncommunicative behaviors (others)**			0.40		3	<.001		24 (3.6)	
	1. Stereotypical behavior	0.21		2		<.001	16 (2.4)		
	2. Injurious behavior to self and others^d^	–0.0003		N/A		.95	2 (0.3)		
	3. Others	0.44		4		<0.001	6 (0.9)		

^a^κ ranges for qualitative interpretation: 0, less than chance; 1, 1.01-0.20; 2, 0.21-0.40; 3, 0.41-0.60; 4, 0.61-0.80; 5, 0.81-0.99 [41].

^b^One score from one rater.

^c^N/A: not applicable.

^d^Needs did not match.

## Discussion

### Principal Results

With the use of location and environmental sensing technologies, we were able to develop ChildSIDE, a mobile app that collects caregivers’ interpretation of the expressive behaviors of children with PIMD/SMID, along with location and environment data. The app was also able to detect and transmit data to the database with above 93% server/API performance, except for iBeacon for which the app had the relatively lowest performance rate of 82.3%. Further, interrater agreement (κ) analysis showed an almost perfect level of agreement between two raters, and we were able to identify and categorize 676 body parts or movements involved in 291 individual behavior data. These analyses showed that expressive behaviors of children with PIMD/SMID were manifested mainly through body and hand movements, and vocalizations.

### App Server/API Performance

Among the location and environment data-sensing technologies that were used, the app had relatively the lowest performance in detecting and transmitting iBeacon data. Although relatively higher, a previous study on the use of the iBeacon system in Friendly VOCA showed the same result [7]. This trend emphasizes the possible problem with the placement of iBeacon devices and not the mobile apps developed. That is, our intervention setup may be problematic, since we placed the iBeacon device approximately 2 meters from the app. Dalkilic et al [32] tested the accuracy of iBeacon devices in sending signals to an app, and found that when iBeacon was close to a mobile phone, the app has difficulty in detecting exactly where the signal is coming from. The authors further explained that the electromagnetic fields or waves generated by mobile phones interfere with those coming from the iBeacon device, resulting in low location accuracy [32]. Their experiments also revealed that when iBeacon devices were placed further away from mobile phones (if there is no radio interference from other iBeacon devices, laptops, or mobile phones), up to 8 meters, the app gave more accurate distance estimations [32]. Aside from this explanation, we also considered that placing iBeacon devices in adjacent rooms led to the difficulty for the app to detect and therefore transmit the iBeacon data to the app database. Thus, we checked if the iBeacon data detected and transmitted by the app to the database originated from the iBeacon installed in the same room. We found that the iBeacon data detected and transmitted by the app to the database were approximately the same (96% similarity) as the data from the iBeacon installed in the same room as the app. This finding is similar to that of Dalkilic et al [32], who examined the effect of walls by placing one iBeacon device and a mobile phone in one room and another iBeacon in an adjacent room. They found that the wall between the two rooms blocked the signals from the iBeacon that was not in the same room as the app [32].

### iBeacon RSSI

Although we acknowledge and plan to address the problems in our intervention setup, specifically with respect to the placement of the iBeacon device relative to its distance from the app, we also assumed that the problem may have been related to the signal strength of the iBeacon device that we used, which was different from that used in our previous work. Paek et al [31] tested three iBeacon devices, and found that the variation in the signal was much too high, and the RSSI values and corresponding signal propagation model varied significantly across iBeacon vendors and mobile platforms. To address this variability, we plan to test different iBeacon devices from different vendors, and choose the best product that fits our mobile platform and the goal of our study in the future. Most importantly, we will also consider an iBeacon device (BLE) company that conforms to the regulations and technical standards of Japan Radio Law (Japan Quality Assurance Association), with US FCC Part 15 and Harmonized European Standard EN300 328 compatibility [30].

### Future Developments

The Friendly VOCA and ChildSIDE apps are part of a holistic system that will enable children to have independent communication and mobility. This initial study confirms that children’s behaviors were manifested mainly through hand and body movements, which provide the structure of an inference smart environment system that can predict children’s needs through speech or movement patterns. Part of our future work plan is to develop models that will make use of the behavior data that were collected in this study, and to construct machine- or deep-learning algorithms to predict children’s needs. Once developed, the model will be used to analyze new behavior data that will be collected using an optical motion camera, as a recently developed technology to capture human movements [42]. The outputs will be analyzed using movement trajectory software, a powerful tool in motor behavior studies [43]. That is, the ChildSIDE app will be connected to the optical motion camera using a common database to enable the continuous and seamless transmission of data. The predictive models will then be passed through the database to our previously developed Friendly VOCA that will produce the specific sound or voice. This will allow smart speakers to respond to the children’s needs either by sending voice commands to home gadgets and appliances (eg, television, lights, or air conditioning system) or to inform the caregivers of the need for support and assistance.

In the future, we will make use of the ChildSIDE app and the optical motion camera in collecting new behavior data to build an automatic and individualized predictive model for each child with PIMD/SMID, or other neurological or physical or motor impairments, resulting in clear provision of an individual-centered, holistic, and smart environment inference system. With this goal in mind, an equally important and interesting line of study in the future is to test its application among adults or older populations with PIMD/SMID or other conditions for communication or delivering rehabilitation interventions.

Although this study was limited to and focused on testing the app’s performance in detecting and transmitting environmental data to the database, it is also noteworthy to consider that weather variables such as humidity and solar radiation were identified as predictors of changes in the emotional and behavioral states of children [9]. Our future work, as part of the inference system that we will develop, will also explore the possibility of whether movements, behaviors, and consequently the needs of children with PIMD/SMID can be predicted using the environmental data (location and weather variables) that were collected in this study.

At present, we only rely on the interpretations of the children’s close caregivers (eg, parents, teachers, therapists) because the children are highly dependent on them for pervasive support in everyday tasks, 24 hours a day [1,4]. These caregivers are more capable of discerning and interpreting the mostly unique behaviors of each child than other people. Thus, we are expecting that our system will help people who are not as close to the children to easily communicate with them and be part of their communication group.

### Strengths and Limitations

One of the main strengths of this study was the inclusion of a relatively high number of children with PIMD/SMID, or severe or profound intellectual disabilities (n=20) compared with similar previous studies, which only had a maximum of two children assessed. This enabled us to perform 105 multiple face-to-face and video-recorded sessions, and collect 371 individual behavior data, which were analyzed and categorized. With the use of the app, this study contributes to the emerging body of evidence in categorizing the expressive behaviors of children with PIMD/SMID, which can be of great help in designing and planning interventions.

Despite these strengths, several limitations of this study may affect the generalizability of our findings. We were able to perform multiple sessions among our target population; however, we only performed these sessions in a school setting. This limits our study in providing a more diverse perspective on children's behaviors, as they have distinctive behaviors and needs at home and toward their immediate family members who they are more familiar with. This will be taken into consideration in our plans of testing the app at home and other locations, which will help to measure the ability of the app in detecting and transmitting behavior, location, and environment data to the app database in a different setting. Most importantly, we also acknowledge that although we strictly adhered to the session protocol, the potential confounding effect of the presence of the investigator or the intervention may have affected the validity of the behavior data collected from the children.

Our findings are also limited to children with PIMD/SMID who are attending special needs schools. Since some children with similar needs attend regular schools or are in health care facilities, we will consider including these settings in our future interventions. Another limitation of our study was the method used to measure the performance rate of the app in detecting and transmitting location and environment data from iBeacon, GPS, ALPS sensors, and OpenWeatherMap API data sources. Although it is ideal to measure the app’s server/API performance by comparing it with other apps that use similar location and environment data-sensing technologies, to the best of our knowledge, no other app has been developed with the same goals and functions as the ChildSIDE app to date. Consequently, we had no other means of measuring this function other than counting the data transmitted and detected by the app to the database.

Our findings on the movements involved in the behaviors of the children with PIMD/SMID were limited to the children recruited in our study and may not represent the population in general; thus, they must be interpreted with caution. Lastly, we consider a potential language limitation on the translation of our data from Japanese to English. Although the data were translated by a bilingual translator, we still consider that there were words in Japanese that did not have an English equivalent or were difficult to translate in English in the same context. This also leads to the limitations on the generalizability of our findings and conclusions, which may only represent children with PIMD/SMID in the Japanese population and could differ in other countries.

### Conclusions

This study confirms that the ChildSIDE app is an effective tool in collecting the behavior of children with PIMD or SMID along with associated GPS location and environment data, as revealed by its high server/API performance rates. However, the app had difficulty in detecting and transmitting short-distance indoor location sensor data from iBeacon devices. This study also adds to the emerging body of evidence for the possibility of categorizing and interpreting the expressive behaviors of children with PIMD/SMID. This emphasizes the need to develop a system that uses motion-capture technology and to develop algorithms using machine or deep learning as part of an individual-centered, holistic, and smart environment inference system to predict the needs of children with PIMD/SMID in the future.

## Appendix

Multimedia Appendix 1 Location, frequency, estimated mean (SD) distance, mean error, and root mean square error (RMSE) to the iBeacon and the ChildSIDE app.

Multimedia Appendix 2 Behavior data collected by ChildSIDE app.

## Abbreviations

API: application programming interface
BLE: Bluetooth low energy
FCC: Federal Communications Commission
PIMD: profound intellectual and multiple disabilities
RSSI: radio signal strength indication
SMID: severe motor and intellectual disabilities
VOCA: voice output communication aid

## References

[1] Nakken H, Vlaskamp C (2007). A need for a taxonomy for profound intellectual and multiple disabilities. J Policy Practice in Intell Disabilities.

[2] Ozawa H, Kato I, Ozaki H, Ishizuka T, Arimoto K, Kimiya S (2007). The present situation of children with psycho-motor disabilities and their parents. No To Hattatsu.

[3] Orelove F, Sobsey D (1991). Educating children with multiple disabilities: A transdisciplinary approach, 2nd edition.

[4] Palisano RJ, Hanna SE, Rosenbaum PL, Russell DJ, Walter SD, Wood EP, Raina PS, Galuppi BE (2000). Validation of a model of gross motor function for children with cerebral palsy. Phys Ther.

[5] Oeseburg B, Dijkstra GJ, Groothoff JW, Reijneveld SA, Jansen DEMC (2011). Prevalence of chronic health conditions in children with intellectual disability: a systematic literature review. Intellect Dev Disabil.

[6] van Timmeren EA, van der Putten AAJ, van Schrojenstein Lantman-de Valk HMJ, van der Schans CP, Waninge A (2016). Prevalence of reported physical health problems in people with severe or profound intellectual and motor disabilities: a cross-sectional study of medical records and care plans. J Intellect Disabil Res.

[7] Karita T (2017). Development of a communication aid app with iOS devices to support children/persons with speech disabilities improvement in obtaining positioning information with iBeacon as near field radio communication technology. J Adv Comput Intell Intell Inform.

[8] Schank R (1977). Scripts, Plans, Goals, and Understanding.

[9] Ciucci E, Calussi P, Menesini E, Mattei A, Petralli M, Orlandini S (2013). Seasonal variation, weather and behavior in day-care children: a multilevel approach. Int J Biometeorol.

[10] Harrison F, Goodman A, van Sluijs EMF, Andersen LB, Cardon G, Davey R, Janz KF, Kriemler S, Molloy L, Page AS, Pate R, Puder JJ, Sardinha LB, Timperio A, Wedderkopp N, Jones AP, on behalf of the ICAD collaborators (2017). Weather and children's physical activity; how and why do relationships vary between countries?. Int J Behav Nutr Phys Act.

[11] Lagacé-Séguin DG (2001). Winter weather go away, come again another day! Meteorology and mothers’ perceptions of children’s emotions during the winter season. Can J Res Early Child Educ.

[12] Lagacé-Séguin DG, d’Entremont Ml (2007). Weathering the preschool environment: affect moderates the relations between meteorology and preschool behaviors. Early Child Dev Care.

[13] VanBuskirk SE, Simpson RL (2013). Meteorological variables and behavior of learners with autism. Focus Autism Other Dev Disabl.

[14] Ashida K, Ishikura K (2013). Creating a category table of expressions in children with severe motor and intellectual disabilities. Journal of School Education.

[15] Maes B, Lambrechts G, Hostyn I, Petry K (2007). Quality-enhancing interventions for people with profound intellectual and multiple disabilities: a review of the empirical research literature. J Intellect Dev Disabil.

[16] Olsson C (2009). Dyadic interaction with a child with multiple disabilities: a system theory perspective on communication. Augment Altern Commun.

[17] Yashaswini R, Manjula R (2016). Pre-symbolic and symbolic communication behaviors of typically developing children (1.6 years) in dyadic communication context using adapted communication complexity scale. IOSR J Human Soc Sci.

[18] Tanaka M, Inui H, Kume S, Maegawa C, Yanagawa C (2000). Student behavior during the teaching and learning process: educating students with profound retardation. Japan J Spec Educ.

[19] Motoda M, Fujita T, Narita S (2002). Communication between residents and staff members in an institution for persons with severe disabilities: using a self-monitoring check sheet. Japan J Spec Educ.

[20] Ashida K, Ishikura K (2015). The understanding of expression for children with severe motor and intellectual disabilities: The analysis using the category table of expressions. Journal of School Education.

[21] Harari GM, Lane ND, Wang R, Crosier BS, Campbell AT, Gosling SD (2016). Using smartphones to collect behavioral data in psychological science: opportunities, practical considerations, and challenges. Perspect Psychol Sci.

[22] Zhang J, Calabrese C, Ding J, Liu M, Zhang B (2017). Advantages and challenges in using mobile apps for field experiments: A systematic review and a case study. Mobile Media Commun.

[23] Njuguna HN, Caselton DL, Arunga GO, Emukule GO, Kinyanjui DK, Kalani RM, Kinkade C, Muthoka PM, Katz MA, Mott JA (2014). A comparison of smartphones to paper-based questionnaires for routine influenza sentinel surveillance, Kenya, 2011-2012. BMC Med Inform Decis Mak.

[24] King JD, Buolamwini J, Cromwell EA, Panfel A, Teferi T, Zerihun M, Melak B, Watson J, Tadesse Z, Vienneau D, Ngondi J, Utzinger J, Odermatt P, Emerson PM (2013). A novel electronic data collection system for large-scale surveys of neglected tropical diseases. PLoS One.

[25] Harari GM, Gosling SD, Wang R, Campbell AT (2020). Capturing situational information with smartphones and mobile sensing methods. Eur J Pers.

[26] Masoud M, Jaradat Y, Manasrah A, Jannoud I (2019). Sensors of smart devices in the Internet of Everything (IoE) era: Big opportunities and massive doubts. J Sensors.

[27] Huawei P9 Lite. Freetel.

[28] Android Studio. Developers.

[29] Huawei nova lite 2. Huawei.

[30] Batteries Beacon. Braveridge.

[31] Paek J, Ko J, Shin H (2016). A measurement study of BLE iBeacon and geometric adjustment scheme for indoor location-based mobile applications. Mobile Inf Syst.

[32] Dalkılıç F, Çabuk UC, Arikan E, Gürkan A (2017). An analysis of the positioning accuracy of iBeacon technology in indoor environments.

[33] What are Broadcasting Power, RSSI and other characteristics of a beacon's signal?. Estimote.

[34] IoT Smart Module Sensor Network Module Evaluation Kit (Development Purposes Only). ALPS ALPINE.

[35] Accuracy and quality of weather data. OpenWeather.

[36] Google Firebase.

[37] Kroesbergen EH, Van Luit JE (2016). Mathematics interventions for children with special educational needs. Remed Spec Educ.

[38] World Medical Association (2013). World Medical Association Declaration of Helsinki: ethical principles for medical research involving human subjects. JAMA.

[39] European Medicines Agency (2002). ICH Topic E6 (R1) Guideline for Good Clinical Practice Step 5 Note for Guidance on Good Clinical Practice. CPMP/ICH/135/95.

[40] Asan O, Montague E (2014). Using video-based observation research methods in primary care health encounters to evaluate complex interactions. Inform Prim Care.

[41] Ahmed R, Robinson R, Elsony A, Thomson R, Squire SB, Malmborg R, Burney P, Mortimer K (2018). A comparison of smartphone and paper data-collection tools in the Burden of Obstructive Lung Disease (BOLD) study in Gezira state, Sudan. PLoS One.

[42] Tirakoat S (2011). Optimized motion capture system for full body human motion capturing case study of educational institution and small animation production.

[43] Blinch J, Kim Y, Chua R (2018). Trajectory analysis of discrete goal-directed pointing movements: How many trials are needed for reliable data?. Behav Res Methods.

