# Supplementary material for: Integrating Behavior of Children with Profound Intellectual, Multiple, or Severe Motor Disabilities With Location and Environment Data Sensors for Independent Communication and Mobility: App Development and Pilot Testing
Source: JMIR Rehabil Assist Technol. 2021 Jun 7;8(2):e28020. doi: 10.2196/28020 (PMC8218217; doi:10.2196/28020)
Supplement: Multimedia Appendix 1 [file rehab_v8i2e28020_app1.docx]

| **iBeacon name** | **Room** | **Specific location** | **Frequency**  **(n = 162)** | **EMD** | **SD** | **ME** | **RMSE** |
| --- | --- | --- | --- | --- | --- | --- | --- |
| Dog | Elementary 3D | White board | 3 | 2.201 | 0.078 | 0.201 | 0.449 |
| Wild boar | Playroom | Above the cleaning supplies closet | 14 | 2.155 | 0.070 | 0.155 | 0.394 |
| Dolphin | Elementary 3C | On the shelf | 18 | 1.946 | 0.192 | 0.054 | 0.233 |
| Bat* | Music Room 1 | Above the control panel | 8 | 2.153 | 0.100 | 0.153 | 0.391 |
| Zebra* | Music Room 1 | Above the control panel | 8 | 2.270 | 0.059 | 0.270 | 0.520 |
| Ladybug | Elementary 3F | Above the control panel | 55 | 2.214 | 0.158 | 0.214 | 0.463 |
| Stork | Elementary 6F | Near the air conditioner | 36 | 2.223 | 0.099 | 0.223 | 0.472 |
| Penguin | Elementary 5C | Above the blackboard | 18 | 2.201 | 0.071 | 0.201 | 0.449 |
| Human | Child Play Room | Edge of the board | 2 | 2.210 | 0.072 | 0.210 | 0.458 |

**Table S1:** Location, frequency, estimated mean distance and SD, and mean error and RMSE to the iBeacon and the ChildSIDE app during the first batch of the session

*The name of the iBeacons were changed to differentiate the data collected by one iBeacon device from two different locations and time.

**Table S2:** Location, frequency, estimated mean distance and SD to the iBeacon and the ChildSIDE app during the second batch of the session

| **iBeacon name** | **Room** | **Specific location** | **Frequency**  **(n = 70)** | **EMD** | **SD** | **ME** | **RMSE** |
| --- | --- | --- | --- | --- | --- | --- | --- |
| Dog | Year 2, Class 4 | On the blackboard | 12 | 2.294 | 0.062 | 0.294 | 0.543 |
| Dolphin | Year 3, Class 4 | On a shelf | 8 | 2.111 | 0.155 | 0.111 | 0.334 |
| Ladybug | Year 3, Class 7 | White board | 36 | 1.984 | 0.150 | 0.016 | 0.126 |
| Stork | Year 2, Class 6 | On the Blackboard | 9 | 2.221 | 0.042 | 0.221 | 0.470 |

**Table S3:** Location, frequency, estimated mean distance and SD to the iBeacon and the ChildSIDE app during the third batch of the session

| **iBeacon name** | **Room** | **Specific location** | **Frequency**  **(n = 37)** | **EMD** | **SD** | **ME** | **RMSE** |
| --- | --- | --- | --- | --- | --- | --- | --- |
| Dog | Year 1, Class 3 | Above the control panel | 15 | 2.247 | 0.066 | 0.247 | 0.497 |
| Dolphin | Year 1, Class 5 | Above the blackboard | 14 | 2.016 | 0.146 | 0.016 | 0.125 |
| Ladybug | Year 1, Class 8 | On the blackboard | 3 | 2.235 | 0.000 | 0.235 | 0.485 |
| Stork | Year 1, Class 7 | On the blackboard | 5 | 2.245 | 0.089 | 0.245 | 0.495 |

Note: EMD = estimated mean distance is the mean distance determined by the RSSI of all the iBeacon devices used in the sessions in relation to the 2-meter actual distance; SD = standard deviation; ME = Mean error is mean difference between the actual distance and the estimated mean distance; RMSE = root mean square error is derived by squaring the differences between actual distance and the estimated distance
