# Supplementary material for: Integrating Behavior of Children with Profound Intellectual, Multiple, or Severe Motor Disabilities With Location and Environment Data Sensors for Independent Communication and Mobility: App Development and Pilot Testing
Source: JMIR Rehabil Assist Technol. 2021 Jun 7;8(2):e28020. doi: 10.2196/28020 (PMC8218217; doi:10.2196/28020)
Supplement: Multimedia Appendix 2 [file rehab_v8i2e28020_app2.docx]

Frequency distribution and percentage of the collected expressive behavior data by the ChildSIDE app.

| Expressive behaviors | n = 291 (%) |
| --- | --- |
| 1. “Yes” | 68 (23.3) |
| 2. Expressing concern | 36 (12.3) |
| 3. “This” | 35 (12.0) |
| 4. Calling teacher | 35 (12.0) |
| 5. “I want to do…” | 22 (7.5) |
| 6. “No” | 17 (5.8) |
| 7. No meaning^a^ | 9 (3.1) |
| 8. Expressing happiness | 8 (2.7) |
| 9. “Delicious” | 5 (1.7) |
| 10. Expressing want to eat or food | 5 (1.7) |
| 11. Being angry | 5 (1.7) |
| 12. “Sleepy” | 5 (1.7) |
| 13. “I did it.” | 4 (1.4) |
| 14. “Goodbye” | 4 (1.4) |
| 15. Showing positive emotions | 4 (1.4) |
| 16. Worried | 4 (1.4) |
| 17. Tired | 4 (1.4) |
| 18. “Good morning” | 3 (1.0) |
| 19. “Fun” | 3 (1.0) |
| 20. “Show me” | 3 (1.0) |
| 21. “I like…” | 3 (1.0) |
| 22. “Here you go /here you are” | 2 (0.7) |
| 23. “No, it's different” | 2 (0.7) |
| 24. “Finished” | 2 (0.7) |
| 25. Thirsty | 1 (0.3) |
| 26. Failed to interpret^b^ | 1 (0.3) |
| 27. “Cold” | 1 (0.3) |
| 28. Expressing hurt or painful | 1 (0.3) |
